# Supplementary material for: Examining the Relationship between Cardiorespiratory Fitness and Body Weight Status: Empirical Evidence from a Population-Based Survey of Adults in Taiwan
Source: ScientificWorldJournal. 2014 Oct 15;2014:463736. doi: 10.1155/2014/463736 (PMC4214098; doi:10.1155/2014/463736)
Supplement: Supplementary file 1 — Tables A1 and A2 present the detailed estimated results of the CRF equation, including the coefficients on the other controlled variables omitted in Table 3 in the main text. As shown in Tables A1 and A2, compared to those who finished primary education or less (the reference group), the adult males and females who obtained junior high school, senior high school, college, and graduate school degree had a lower CRF score. The age of the adult males and females was found to be negatively related to their CRF while the income level was positively related to the CRF. In addition, the job categories, urbanization levels, and geographic locations were significantly associated with the CRF. [file 463736.f1.pdf]

Hindawi Publishing Corporation
The Scientific World Journal
Volume 2014, Article ID 463736, 7 pages
http://dx.doi.org/10.1155/2014/463736

Research Article

Examining the Relationship between Cardiorespiratory Fitness and Body Weight Status: Empirical Evidence from a Population-Based Survey of Adults in Taiwan

Tai-Hsiung Hung,[1] Pei-An Liao,[2] Hung-Hao Chang,[3] Jiun-Hao Wang,[4] and Min-Chen Wu[5]

[1] *Undergraduate Academic Affairs Division, National Taiwan University, Taipei 10617, Taiwan*
[2] *Department of Economics, Shih Hsin University, Taipei 11645, Taiwan*
[3] *Department of Agricultural Economics, National Taiwan University, Taipei 10617, Taiwan*
[4] *Department of Bio-Industry Communication and Development, National Taiwan University, No. 1, Roosevelt Road Section 4, Taipei 10617, Taiwan*
[5] *Office of Physical Education, Chung Yuan Christian University, Chung-Li 32023, Taiwan*

Correspondence should be addressed to Jiun-Hao Wang; wangjh@ntu.edu.tw

Received 8 July 2014; Revised 28 August 2014; Accepted 15 September 2014; Published 15 October 2014

Academic Editor: Denes Molnár

Copyright © 2014 Tai-Hsiung Hung et al. This is an open access article distributed under the Creative Commons Attribution License, which permits unrestricted use, distribution, and reproduction in any medium, provided the original work is properly cited.

Background. Cardiovascular disease is the number one cause of death worldwide. Meanwhile, obesity has been recognized as a global epidemic. This study aims to examine the extent to which cardiorespiratory fitness is associated with body mass among adult males and females in Taiwan. *Materials and Methods*. A nationally representative dataset consisting of 68,175 adults aged 18–60, including 31,743 males and 36,432 females, was used. Several multivariate regression models were used to investigate the relationship between cardiorespiratory fitness and body weight status, after controlling for adults' sociodemographic status. *Results*. A one-unit increase in the BMI lowered the cardiorespiratory fitness score by 0.316 and 0.368 points for adult males and females, respectively. Among adult males, compared to those of normal weight, adult males who were underweight, overweight, or obese had a lower cardiorespiratory fitness score by 1.287, 0.845, and 3.353 points, respectively. Similar results could be found in female samples. *Conclusion*. The overweight and obese adults had much lower levels of cardiorespiratory fitness as compared to their normal weight counterparts. Given the upward trend in the prevalence of overweight and obesity, it is important to help overweight and obese people to become more fit and reach their healthy weight.

1. Introduction

The cardiovascular system consists of the heart, blood vessels, and blood. It provides several essential functions necessary for life, such as transporting oxygen and nutrients, removing carbon dioxide and wastes, fighting disease, and maintaining body temperature [1]. Cardiovascular disease (CVD), the number one cause of death worldwide, refers to any disease that affects the cardiovascular system. It was estimated that 17.5 million people died due to CVD in 2012, representing 30% of all global deaths; of these deaths, coronary heart disease and stroke were the two leading causes [2].

Many risk factors have been found to be associated with CVD, such as hypertension, diabetes, unhealthy diets, smoking, harmful use of alcohol, obesity, and low cardiorespiratory fitness (CRF) [3, 4]. Among these risk factors, obesity has received particular attention as obesity has been recognized as a global epidemic. The number of overweight/obese adults worldwide was 2.1 billion in 2013, compared with 857 million in 1980 [5]. In the United States, more than two-thirds of adults are considered to be overweight or obese [6]. Similar to many other countries, Taiwan's population has experienced a rising trend of excess weight and obesity over the decades as well. In 2013, overweight (including obesity) rates were 45.9% and 33.1% among men and women, respectively, in Taiwan [7].

Compared with obesity, the impact of CRF on human health has often been ignored, even though it appears to be

one of the most important determinants of overall health status and a powerful predictor of CVD mortality and morbidity [3, 8, 9]. CRF refers to the ability of the circulatory and respiratory systems to efficiently supply oxygen to the working muscles during sustained physical activity. It has been documented that CRF can be measured using a variety of modes of exercise, such as the treadmill, cycle ergometer, or a step test [9, 10].

Given the fact that CRF and obesity are two important risk factors for CVD, it is of interest to examine the relationship between CRF and obesity. Doing so will allow for a better understanding of the potential mechanisms that mediate the links between CRF, obesity, and CVD. Numerous studies have found that CRF is attenuated with increasing body mass [11–16]. For example, using 1,003 sixth graders in Michigan, USA, Eagle et al. [14] found that, compared to their nonobese peers, obese students demonstrated lower CRF levels. These previous studies, however, mainly focused on children and adolescents; not many studies so far have investigated adults, and among these few studies, the focus is mainly on adults in Western societies, such as the United States and Italy [17–19]. The existing studies found a negative relationship between CRF and body mass.

The objective of the present study was to examine the extent to which CRF is associated with body mass among male and female adults in Taiwan. Our study contributes to the previous literature mainly on three fields. First, a large-scale, population-based, and nationally representative sample of the adults in Taiwan was used. In contrast with most previous studies that used small-scale samples or restricted subjects to specific clinics, schools, or regions, the dataset used in this analysis was unique. Second, the participants' CRF, height, and weight were objectively measured via health-center or test-station visits, and thus the measures of CRF and body weight status in our dataset were free of self-reported bias. Lastly, this study focused on Asian adults, who make up the world's largest population but have not been extensively discussed before in the context of the relationship between CRF and body mass.

2. Materials and Methods

2.1. Data. The data used in this analysis are the physical fitness profiles of Taiwanese adults aged 18–60. Aiming at reducing the risks of CVD and monitoring the physical fitness status of Taiwan's adult population, the Sports Administration, Ministry of Education (MOE), Taiwan, conducted a nationwide physical fitness test, primarily targeted on CRF and body mass, for the adult population in 2012. Participants were selected based on a stratified multistage sampling scheme in order to obtain a nationally representative sample. Each selected participant was examined using physical fitness tests and a standardized face-to-face interview in the nearby health center or test station in each county. This dataset contained unique information on objectively measured CRF, height, and weight. In addition, information pertaining to participants' sociodemographic characteristics was also documented. In total, the dataset included 70,042 adults aged 18–60. After further deleting a small number of observations with missing data, the final sample consisted of 68,175 adults, of whom 31,743 were male and 36,432 were female.

2.2. Measures

2.2.1. Cardiorespiratory Fitness. Each participant's physical fitness tests were conducted by physical education instructors in the health center or test station of each county. The physical education instructors were required to participate in a three-day training camp hosted by the MOE to learn detailed instructions on the proper procedure for the tests. Only those who passed the certification exams were qualified to carry out adult participants' physical fitness tests.

The Harvard step test [20] was used for the evaluation of CRF. The equipment required included a 35 cm high platform, stopwatch, and metronome. The metronome was set at 96 beats per minute (4 clicks being equal to one-step cycle) for a stepping rate of 24 steps per minute. The participants stepped up and down on the platform for 3 minutes or until they experienced exhaustion. In time with the beat, the participant stepped one foot up on the platform (1st beat), stepped up with the second foot (2nd beat), stepped down with one foot (3rd beat), and stepped down with the other foot (4th beat). The participant immediately sat down on completion of the test, and the total number of heartbeats was counted between 1 and 1.5, 2 and 2.5, and 3 and 3.5 minutes after finishing. The fitness index score was used to reflect CRF and was determined by the following equation:

$$\text{CRF} = \frac{\text{test duration in seconds (i.e., 180 seconds)} \times 100}{2 \times (\text{sum of 3 measures of heart beats})}. \quad (1)$$

The faster the participant's heart rate returned to resting, the better his/her CRF was. Hence, a higher value of the score indicated a better CRF performance.

2.2.2. Body Mass Index. Each participant's height and weight were measured by physical education instructors. We calculated the Body Mass Index (BMI) defined as weight in kilograms divided by the square of height in meters: kg/m^2. Based on the official cut-off points for different weight statuses defined by the Ministry of Health and Welfare, Taiwan, participants were recognized as underweight if $BMI < 18.5$, of normal weight if $18.5 \le BMI < 24$, overweight if $24 \le BMI < 27$, and obese if $27 \le BMI$ [21].

2.2.3. Other Determinants. Information on participants' soc iodemographic characteristics was also collected in this dataset. We separated the available information into several categories, including gender, education level, age, individual monthly income, occupation, urbanization level, and geographic location. These variables were used as controls (i.e., exogenous variables in the multivariate linear regression models) when we examined the relationship between CRF and body weight status.

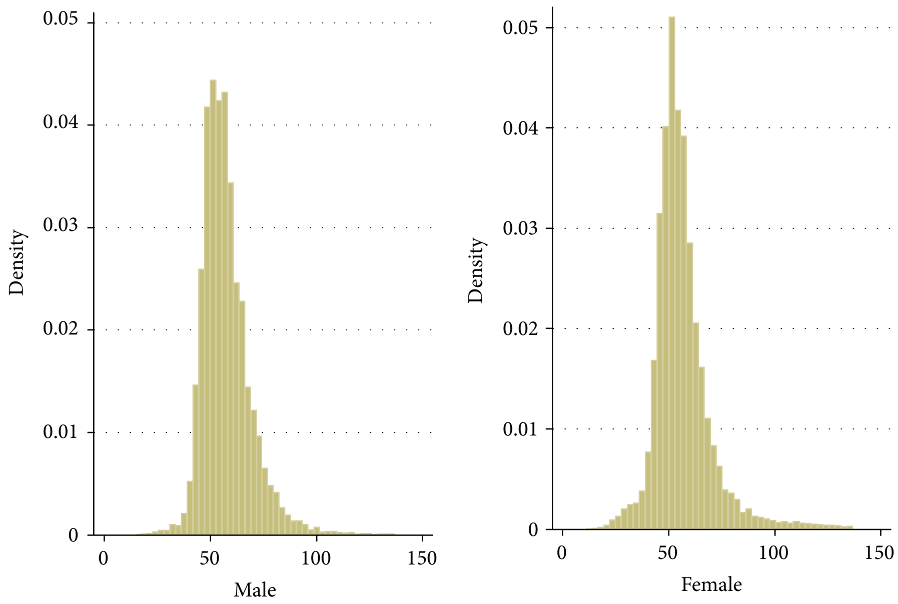

FIGURE 1: Sample distribution of the CRF score by gender. Sample information: males: min (14.70), max (136.92); females: min (11.03), max (136.82).

2.3. Statistical Analysis. Two multivariate linear regression models were used to examine the relationship between CRF and body weight status. The BMI as a continuous variable was used to capture each participant's body mass in the first regression model. Assuming that a continuous variable y_i indicates the CRF index score of the participant i, and the vector x_i represents a set of exogenous variables correlated with the fitness score, the linear regression model can be specified as

$$y_i = \alpha + \gamma \times \mathrm{BMI}_i + \beta' x_i + \varepsilon_i, \quad (2)$$

where γ and β are parameters and ε_i is the random error term. We are interested in the parameter γ that captures the correlation between CRF and BMI, after controlling for the other sociodemographic characteristics. The consistent estimates of $\widehat{\gamma}$ and $\widehat{\beta}$ can be obtained by using the ordinary least squares (OLS) method.

Since the relationship between CRF and BMI may not be linear, we further estimated the second regression model by categorizing weight status into four groups: underweight, normal weight, overweight, and obesity. When normal weight status is treated as the reference group, the estimated equation can be specified as

$$\begin{aligned} y_i = \alpha &+ \gamma_1 \times \mathit{Underweight}_i + \gamma_2 \times \mathit{Overweight}_i \\ &+ \gamma_3 \times \mathit{Obesity}_i + \beta' x_i + \varepsilon_i, \end{aligned} \quad (3)$$

where *Underweight*, *Overweight*, and *Obesity* are dummy variables equal to 1 for individuals who are underweight, overweight, and obese, respectively. In (3), the coefficients γ_1, γ_2, and γ_3 then, respectively, capture the differences in CRF between the underweight, overweight, and obese adults compared to those who are of normal weight (the reference group). To further capture the potential gender differences, we estimated (3) separately for male and female adults. All of the empirical analyses were conducted using STATA version 13.

TABLE 1: CRF score stratified by gender and weight status.

	Male	Female
All samples	57.59 (11.47)	56.52 (13.65)
By weight status		
Underweight	57.48 (10.45)	57.46 (14.46)
Normal weight	58.64 (10.69)	57.22 (13.86)
Overweight	57.75 (12.21)	55.70 (12.46)
Obesity	55.12 (12.39)	52.38 (14.46)

(·) reports the standard deviation.

3. Results

Figure 1 visually presents how the CRF measurement is distributed among adult males and females. In addition, the sample means and standard deviations of the CRF score by gender and weight status are reported in Table 1. The average CRF score of adult males is slightly higher than that of females (57.59 versus 56.52). The CRF score also differs for adults with different weight categories. Adult males of normal weight have the highest score (58.64). In contrast, the lowest score is found among adult males who are obese (55.12). A slightly different finding is revealed among adult females. The highest score is found among adult females who are underweight (57.46) while the lowest score is shown among those who are obese (52.38). The results in Table 1 provide some preliminary evidence that the CRF score may be different among adults with different weight status and

Table 2: Definitions of study variables and summary statistics of participants.

Variable	Definition	Male		Female	
		Mean	SD	Mean	SD
CRF	Fitness index score reflecting cardiorespiratory fitness	57.59	11.47	56.52	13.65
BMI	Body Mass Index (kg/m^2)	24.26	3.85	22.30	3.61
Underweight	If they are underweight (BMI < 18.5), then =1	0.05	0.21	0.11	0.31
Normal weight	If they are of normal weight (18.5 ≤ BMI < 24), then =1	0.45	0.50	0.64	0.48
Overweight	If they are overweight (24 ≤ BMI < 27), then =1	0.29	0.45	0.16	0.36
Obesity	If they are obese (27 ≤ BMI), then =1	0.21	0.41	0.10	0.30
Primary	If they finished primary education or less (=1)	0.02	0.15	0.03	0.18
Junior	If they finished junior high school (=1)	0.04	0.20	0.05	0.22
Senior	If they finished senior high school (=1)	0.17	0.37	0.17	0.38
College	If they have college degree (=1)	0.67	0.47	0.69	0.46
Graduate	If they have graduate school diploma (=1)	0.10	0.30	0.05	0.23
Age_1820	If age is between 18 and 20 (=1)	0.28	0.45	0.26	0.44
Age_2130	If age is between 21 and 30 (=1)	0.23	0.42	0.23	0.42
Age_3140	If age is between 31 and 40 (=1)	0.20	0.40	0.22	0.42
Age_4150	If age is between 41 and 50 (=1)	0.16	0.37	0.18	0.38
Age_5160	If age is between 51 and 60 (=1)	0.13	0.38	0.10	0.39
Income 1	If their monthly income is ≤NT$20,000 (=1)	0.44	0.50	0.50	0.50
Income 2	If their monthly income is NT$20,001~NT$40,000 (=1)	0.27	0.44	0.35	0.48
Income 3	If their monthly income is NT$40,001~NT$60,000 (=1)	0.19	0.39	0.12	0.33
Income 4	If their monthly income is >NT$60,000 (=1)	0.10	0.30	0.03	0.18
Job 1	If they work for educational job (=1)	0.04	0.20	0.07	0.26
Job 2	If they work for financial sector (=1)	0.02	0.13	0.02	0.14
Job 3	If they work for agricultural sector (=1)	0.01	0.12	0.01	0.08
Job 4	If they work for service sector (=1)	0.10	0.30	0.15	0.36
Job 5	If they are students (=1)	0.31	0.46	0.27	0.44
Job 6	If they work for high technology sector (=1)	0.04	0.19	0.02	0.15
Job 7	If they work for government sector (=1)	0.17	0.38	0.10	0.30
Job 8	If they work for media press (=1)	0.00	0.05	0.00	0.04
Job 9	If they work for business sector (=1)	0.03	0.18	0.03	0.17
Job 10	If they work for medical or health sector (=1)	0.03	0.17	0.08	0.27
Job 11	If they work for construction sector (=1)	0.09	0.28	0.04	0.19
Job 12	If they work for other sectors (=1)	0.16	0.37	0.21	0.41
City	If they live in metropolitan area (=1)	0.29	0.45	0.29	0.45
North	If they live in Northern Taiwan (=1)	0.33	0.47	0.34	0.47
Center	If they live in Central Taiwan (=1)	0.18	0.38	0.18	0.38
South	If they live in Southern Taiwan (=1)	0.39	0.49	0.40	0.49
East	If they live in Eastern Taiwan (=1)	0.11	0.31	0.09	0.28
Sample		31,743		36,432	

those who are obese may demonstrate the lowest CRF score. These conclusions, however, are not necessarily justified, inasmuch as the possible differences in the characteristics between groups have not yet been controlled.

To quantify the relationship between the CRF score and body weight status among the adults, we made estimates using two multivariate regression models, using the continuous BMI values and different categories of body weight status, respectively. Both regression models controlled for the sociodemographic variables defined in Table 2. As shown in Table 2, the average BMI of adult males and females was 24.26 and 22.30 (kg/m^2), respectively. The percentage of normal weight men and women was 45% and 64%, respectively. 67% of men and 69% of women earned a college degree. The majority (71%) of participants ranged in age from 18 to 40 years. 44% of men and 50% of women had monthly income ≤ NT$20,000 (US$670). 31% of men and 27% of women were students (Job 5). 29% of male and female participants were living in metropolitan areas. 39% of men and 40% of women were living in Southern Taiwan.

The estimation results are presented in Panels A and B of Table 3, respectively. As shown in Panel A of Table 3,

Table 3: Estimation of the CRF equations.

Variable	Male		Female	
	Coefficient	SE	Coefficient	SE
		Panel A		
BMI	-0.316^{***}	0.017	-0.368^{***}	0.019
Other controls	Yes		Yes	
Sample	31,741		36,424	
		Panel B		
Underweight	-1.287^{***}	0.304	−0.411	0.321
Overweight	-0.845^{***}	0.154	-1.088^{***}	0.188
Obesity	-3.353^{***}	0.170	-4.645^{***}	0.224
Other controls	Yes		Yes	
Sample	31,741		36,424	

The symbols *** indicates significance at 1% level.
The ordinary least squares (OLS) method was used for model estimations.
Table 2 lists the definitions and sample statistics of other controlled variables.

the participants' CRF score was negatively associated with the BMI regardless of gender. However, it is evident that the negative effect was greater among adult females. Our results indicated that a one-unit increase in the BMI would lower the CRF score by 0.316 and 0.368 points for adult males and females, respectively, given other factors being equal.

Panel B of Table 3 presents the estimation results that used different body weight status instead of using BMI as the key explanatory variables in the CRF equation. In general, regardless of gender, adults who were underweight, overweight, or obese had a lower CRF score, compared to those whose weight was normal. Among male adults, our results showed that, compared to those whose weight was normal, adult males who were underweight, overweight, or obese had a lower CRF score by 1.287, 0.845, and 3.353 points, respectively. Among adult females, those who were overweight and obese, respectively, had a lower CRF score by 1.088 and 4.645 points, compared to adult females whose weight was normal. Table 3 in the main text omitted the coefficients on the other controlled variables, as those are not directly relevant to our analysis. However, since the coefficients may be of general interest, please refer to the Supplementary Material for more details.

4. Discussion

Our study showed that overweight and obese male and female adults had substantially reduced CRF. There are several possible biological mechanisms to explain our findings. First of all, overweight and obese people tend to have a lower proportion of type I and a higher proportion of type II fibers in their muscle, resulting in reduced oxygen uptake [22, 23]. Not uptaking a sufficient amount of oxygen for hyperactive body musculature would decrease the CRF performance. In addition, excess body weight is likely to impair cardiorespiratory functions and cardiac mechanical efficiency for a given workload [24]. Lastly, increased body mass is associated with decreased exercise tolerance and aerobic capacity [25].

Like being overweight and obese, being underweight decreases CRF performance among male adults as well. According to K. Harada and Y. Harada [26], being underweight has a negative effect on cardiovascular functions. Lower BMI is associated with left ventricular hypotrophy, which may lead to lower levels of CRF. No significant differences, however, were found between underweight and normal weight adult females. Biological gender differences related to heart size and functions led to women having lower CRF abilities [1]. In addition, adult females in Taiwan were found to be less likely to exercise regularly [27]. This may explain why the CRF among underweight adult females was not significantly lower, compared to that among normal weight adult females in the reference group.

Given the fact that our findings indicate that being overweight and obese was associated with substantially lower levels of CRF in both men and women, the policy implications we suggested are relatively straightforward. Health care providers should strongly encourage overweight and obese individuals to lose weight by increasing regular physical activity and decreasing daily caloric intake. Policies to promote the importance of regular physical activity and healthy diet should be implemented. For example, the government should create exercise-friendly environments that include not only building new sports centers but also integrating residents, communities, schools, and sports associations to encourage more residents to exercise. For the working people, the government can encourage companies to implement healthy-eating policies by subsidizing food purchases. Moreover, underweight individuals, particularly underweight men, should be another focus of interventions. Possible treatments, such as increasing caloric intake, providing nutritional supplements, and doing more weight lifting exercises, should be advised [28].

Two potential research limitations of this study should be mentioned. First, BMI is the only measure to assess body weight status. Due to data limitation, we did not have further information on participants' body composition (e.g., the density of fat and fat-free mass and waist circumference) that is

likely to be associated with CRF. In addition, health status and dietary patterns, which could possibly relate to CRF, were not available in the dataset. Our findings could be stronger, based on additional collected data. Second, this study employed a cross-sectional design. Therefore, the interpretation of our findings should be regarded with caution because we can only explore the association between CRF and body weight status. If panel data become available, it will be interesting to further address the causality issue.

5. Conclusions

The objective of this study is to examine the relationship between CRF and body weight status among adults. Using a unique adult sample in Taiwan, we found that overweight and obese adults had much lower levels of CRF, compared to their normal weight counterparts. Given the upward trend in the prevalence of overweight and obesity, it is important to help overweight and obese people to become more fit and reach their healthy weight in order to improve the CRF performance and reduce the risk of CVD.

Conflict of Interests

The authors declare that there is no conflict of interests regarding the publication of this paper.

Acknowledgments

The authors appreciate the Sports Administration, Ministry of Education (MOE), Taiwan, for making the confidential data available to them. The findings of this study do not reflect their views and the authors accept any remaining errors.

References

[1] P. I. Aaronson, J. P. T. Ward, and M. J. Connolly, *The Cardiovascular System at a Glance*, Wiley, Hoboken, NJ, USA, 4th edition, 2012.

[2] World Health Organization, "The top 10 causes of death," 2014, http://www.who.int/mediacentre/factsheets/fs310/en/index2.html.

[3] S. N. Blair, J. B. Kampert, H. W. Kohl III et al., "Influences of cardiorespiratory fitness and other precursors on cardiovascular disease and all-cause mortality in men and women," *Journal of the American Medical Association*, vol. 276, no. 3, pp. 205–210, 1996.

[4] World Health Organiztion, "Global health risks: Mortality and burden of disease attributable to selected major risks," 2009, http://www.who.int/healthinfo/global_burden_disease/GlobalHealthRisks_report_full.pdf.

[5] M. Ng, T. Fleming, M. Robinson et al., "Global, regional, and national prevalence of overweight and obesity in children and adults during 1980–2013: a systematic analysis for the Global Burden of Disease Study 2013," *The Lancet*, vol. 384, no. 9945, pp. 766–781, 2014.

[6] C. L. Ogden, M. D. Carroll, B. K. Kit, and K. M. Flegal, "Prevalence of childhood and adult obesity in the United States, 2011-2012," *The Journal of the American Medical Association*, vol. 311, no. 8, pp. 806–814, 2014.

[7] Health Promotion Administration, Ministry of Health and Welfare, The results of the 2013 Nutrition and Health Survey in Taiwan, 2014, http://www.hpa.gov.tw/Bhpnet/Web/News/News.aspx?No=201402280001.

[8] S. Kodama, K. Saito, S. Tanaka et al., "Cardiorespiratory fitness as a quantitative predictor of all-cause mortality and cardiovascular events in healthy men and women: a meta-analysis," *Journal of the American Medical Association*, vol. 301, no. 19, pp. 2024–2035, 2009.

[9] D.-C. Lee, E. G. Artero, X. Sui, and S. N. Blair, "Mortality trends in the general population: the importance of cardiorespiratory fitness," *Journal of Psychopharmacology*, vol. 24, no. 4, pp. 27–35, 2010.

[10] J. Watkins, "Step tests of cardiorespiratory fitness suitable for mass testing," *British Journal of Sports Medicine*, vol. 18, no. 2, pp. 84–89, 1984.

[11] J. M. Sorof, T. Poffenbarger, K. Franco, L. Bernard, and R. J. Portman, "Isolated systolic hypertension, obesity, and hyperkinetic hemodynamic states in children," *Journal of Pediatrics*, vol. 140, no. 6, pp. 660–666, 2002.

[12] J. Mota, L. Flores, J. C. Ribeiro, and M. P. Santos, "Relationship of single measures of cardiorespiratory fitness and obesity in young schoolchildren," *The American Journal of Human Biology*, vol. 18, no. 3, pp. 335–341, 2006.

[13] T. P. Singh, J. Rhodes, and K. Gauvreau, "Determinants of heart rate recovery following exercise in children," *Medicine and Science in Sports and Exercise*, vol. 40, no. 4, pp. 601–605, 2008.

[14] T. F. Eagle, R. Gurm, C. S. Goldberg et al., "Health status and behavior among middle-school children in a midwest community: what are the underpinnings of childhood obesity?" *The American Heart Journal*, vol. 160, no. 6, pp. 1185–1189, 2010.

[15] S. Esmaeilzadeh and K. Ebadollahzadeh, "Physical fitness, physical activity and sedentary activities of 7 to 11 year old boys with different body mass indexes," *Asian Journal of Sports Medicine*, vol. 3, no. 2, pp. 105–112, 2012.

[16] D. Choudhuri, S. Choudhuri, and M. Aithal, "Relationship between cardiovascular function and markers of adiposity in young female subjects," *International Journal of Medicine and Public Health*, vol. 3, no. 2, pp. 161–164, 2014.

[17] A. Salvadori, P. Fanari, P. Palmulli et al., "Cardiovascular and adrenergic response to exercise in obese subjects," *Journal of Clinical and Basic Cardiology*, vol. 2, no. 2, pp. 229–236, 1999.

[18] C. R. Cole, J. M. Foody, E. H. Blackstone, and M. S. Lauer, "Heart rate recovery after submaximal exercise testing as a predictor of mortality in a cardiovascularly healthy cohort," *Annals of Internal Medicine*, vol. 132, no. 7, pp. 552–555, 2000.

[19] Y. J. Cheng, M. S. Lauer, C. P. Earnest et al., "Heart rate recovery following maximal exercise testing as a predictor of cardiovascular disease and all-cause mortality in men with diabetes," *Diabetes Care*, vol. 26, no. 7, pp. 2052–2057, 2003.

[20] L. Brouha, C. W. Health, and A. Graybiel, "Step test simple method of measuring physical fitness for hard muscular work in adult men," *Review of Canadian Biology*, vol. 2, pp. 86–92, 1943.

[21] Health Promotion Administration-Ministry of Health and Welfare-Taiwan, "Body mass index test," 2012, http://health99.hpa.gov.tw/OnlinkHealth/Onlink_BMI.aspx.

[22] A. H. Kissebah and G. R. Krakower, "Regional adiposity and morbidity," *Physiological Reviews*, vol. 74, no. 4, pp. 761–811, 1994.

[23] C. J. Tanner, H. A. Barakat, G. Lynis Dohm et al., "Muscle fiber type is associated with obesity and weight loss," *American Journal of Physiology—Endocrinology and Metabolism*, vol. 282, no. 6, pp. E1191–E1196, 2002.

[24] J. A. Dempsey, W. Reddan, B. Balke, and J. Rankin, "Work capacity determinants and physiologic cost of weight-supported work in obesity," *Journal of applied physiology*, vol. 21, no. 6, pp. 1815–1820, 1966.

[25] A.-C. Norman, B. Drinkard, J. R. McDuffie, S. Ghorbani, L. B. Yanoff, and J. A. Yanovski, "Influence of excess adiposity on exercise fitness and performance in overweight children and adolescents," *Pediatrics*, vol. 115, no. 6, pp. e690–e696, 2005.

[26] K. Harada and Y. Harada, "Childhood underweight affects left ventricular geometry and function," 2012, Circulation, http://circ.ahajournals.org/cgi/content/meeting_abstract/126/21_MeetingAbstracts/A9046.

[27] Sports Administration, Ministry of Education, Survey of Sport City, 2013, http://www.sa.gov.tw/wSite/ct?xItem=3258&ctNode=300&mp=11.

[28] L. Tunjic, *Obesity, Gravity, Strength and Balance*, Lulu, Raleigh, NC, USA, 2005.

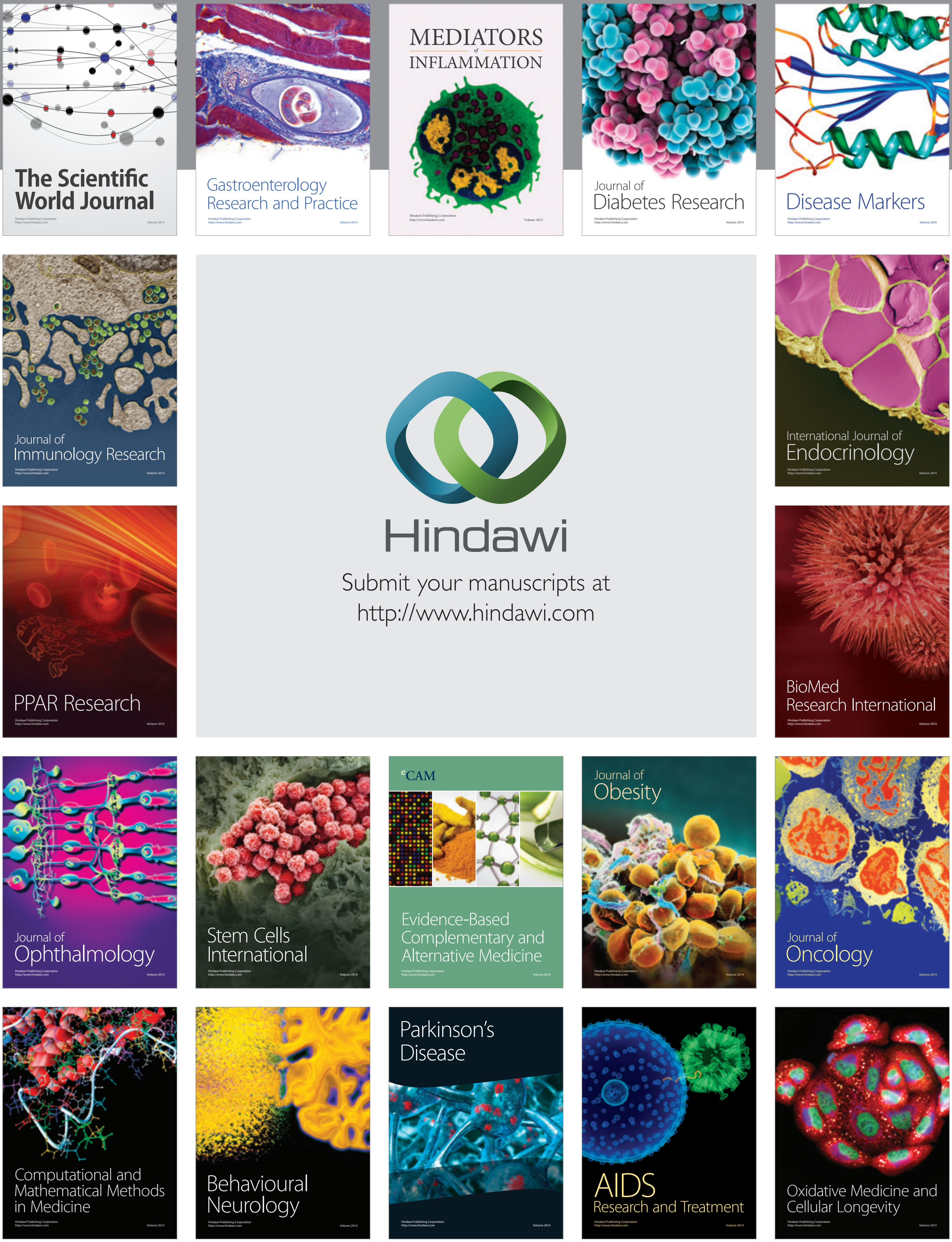
The Scientific World Journal
Gastroenterology Research and Practice
MEDIATORS of INFLAMMATION
Journal of Diabetes Research
Disease Markers
Journal of Immunology Research
Hindawi
Submit your manuscripts at
http://www.hindawi.com
International Journal of Endocrinology
PPAR Research
BioMed Research International
Journal of Ophthalmology
Stem Cells International
eCAM
Evidence-Based Complementary and Alternative Medicine
Journal of Obesity
Journal of Oncology
Computational and Mathematical Methods in Medicine
Behavioural Neurology
Parkinson's Disease
AIDS Research and Treatment
Oxidative Medicine and Cellular Longevity
